# Genetic Diversity and Domestication Footprints of Chinese Cherry [*Cerasus pseudocerasus* (Lindl.) G.Don] as Revealed by Nuclear Microsatellites

**DOI:** 10.3389/fpls.2018.00238

**Published:** 2018-02-27

**Authors:** Jing Zhang, Tao Chen, Yan Wang, Qing Chen, Bo Sun, Ya Luo, Yong Zhang, Haoru Tang, Xiaorong Wang

**Affiliations:** ^1^Institute of Pomology and Olericulture, Sichuan Agricultural University, Ya’an, China; ^2^College of Horticulture, Sichuan Agricultural University, Ya’an, China

**Keywords:** approximate Bayesian computation, Chinese cherry [*Cerasus pseudocerasus* (Lindl.) G.Don], genetic bottlenecks, independent domestication, microsatellite markers

## Abstract

Chinese cherry [*Cerasus pseudocerasus* (Lindl.) G.Don] is a commercially important fruit crop in China, but its structure patterns and domestication history remain imprecise. To address these questions, we estimated the genetic structure and domestication history of Chinese cherry using 19 nuclear microsatellite markers and 650 representative accessions (including 118 *Cerasus* relatives) selected throughout their natural eco-geographical distributions. Our structure analyses detected no genetic contribution from *Cerasus* relatives to the evolution history of Chinese cherry. A separate genetic structure was detected in wild Chinese cherries and rough geographical structures were observed in cultivated Chinese cherries. One wild (wild Chinese cherry, WC) and two cultivated (cultivated Chinese cherry, CC_1_ and CC_2_) genetic clusters were defined. Our approximate Bayesian computation analyses supported an independent domestication history with two domestication events for CC_1_ and CC_2_, happening about 3900 and 2200 years ago, respectively. Moderate loss of genetic diversity, over 1000-year domestication bottlenecks and divergent domestication in fruit traits were also detected in cultivated Chinese cherries, which is highly correlated to long-term clonal propagation and different domestication trends and preferences. Our study is the first to comprehensively and systematically investigate the structure patterns and domestication history for Chinese cherry, providing important references for revealing the evolution and domestication history of perennial woody fruit trees.

## Introduction

Domestication is a complex evolutionary process in which human activities lead domesticated crops to phenotypically and genetically diverge from their wild ancestors (Michael and Dorian, 2009). Recent plant domestication by human beings began about 12,000 years ago, when our ancestors domesticated the main food, fruit, and ornamental crops in current human society (Rachel et al., 2012; Rachel and Michael, 2013). The Rosaceae family includes numerous perennial woody fleshy fruits [apple (*Malus pumila* Mill.), pear (*Pyrus communis* L.), peach (*Amygdalus persica* L.), apricot (*Armeniaca vulgaris* Lam.), and plum (*Prunus salicina* Lindl.)] that have an extraordinary range of variations in the sizes and shapes of fleshy fruits and seeds due to human domestication efforts (Joseph, 2017; Xiang et al., 2017). Therefore, the fruit crops of Rosaceae family are excellent materials for investigating the domestication history and phenotypic divergences of perennial woody fruit trees.

Chinese cherry [*Cerasus pseudocerasus* (Lindl.) G.Don] belongs to the genus *Cerasus* of the Rosaceae family, and is a hermaphrodite perennial woody fruit crop with high levels of inbreeding rate and moderately long juvenile phase (3–6 years) (Yü and Li, 1986). It is an economically and culturally important fruit crop that has been cultivated for more than 3000 years in China (Yü, 1979; Liu et al., 2008). Chinese cherry is thought to have wide natural distributions in Southwest China (SWC) (Yü, 1979), and now has been broadly distributed in Longmenshan Fault Zones (LFZ), Yungui Plateau, Qinling Mountains (QLM) and North China Plain (Huang et al., 2013; Chen et al., 2016), exhibiting considerable genetic and phenotypic variations among eco-geographic regions (Chen et al., 2015; Liu et al., 2016). Beautiful flowers, crystal-clear fruit appearances, wide adaptability and intensive pest/disease resistance make it an important crop in the rural tourist industry of China (Chen et al., 2016).

In recent years, we have mainly estimated the genetic diversity and population structure of Chinese cherry using chloroplast and nuclear DNA sequences, and tried to explore the origin and domestication of this fruit crop (Chen et al., 2013, 2014). However, to date, we have not obtained the overall view of the genetic structure and domestication history of Chinese cherry because uniparentally inherited DNA sequences have limited power to estimate the intraspecies polymorphism and bidirectional gene flow of Chinese cherry. On the other hand, no molecular research addressed whether the divergent phenotypes in fruits and seeds of cultivated Chinese cherries are significantly associated with domestication. Therefore, the core questions about the genetic structure and domestication history of Chinese cherry remained unsolved in previous studies.

Approximate Bayesian computation (ABC) is one of the most powerful methods for conducting the parameter estimation and model selection with microsatellites data, which has been successfully used to infer the domestication history in many plant species (Cornille et al., 2012; Yuan et al., 2014; Dussert et al., 2015). *Q*_ST_–*F*_ST_ comparison conducted with molecular and phenotypic makers also provides a popular means of investigating the main factors of the observed phenotypic differentiation for researchers (Leinonen et al., 2013). Therefore, in this study, we selected 704 Chinese cherry accessions and their *Cerasus* relatives throughout their natural distributions, and used 20 polymorphic nuclear microsatellite loci developed from Chinese cherry genome to explore the structure patterns and domestication history of Chinese cherry with ABC analysis and *Q*_ST_–*F*_ST_ comparison. Our aims were to (i) investigate the genetic structure of Chinese cherry at species level, (ii) explore whether *Cerasus* relatives contribute to the evolution and domestication of Chinese cherry, (iii) estimate the domestication bottlenecks within cultivated Chinese cherry, the number of domestication event(s), and the divergent time between wild and cultivated Chinese cherry accessions, and (iv) clarify whether the fruit and seed traits of cultivated Chinese cherries are significantly associated with the domestication activities.

## Materials and Methods

### Samples and Location

**FIGURE 1:**
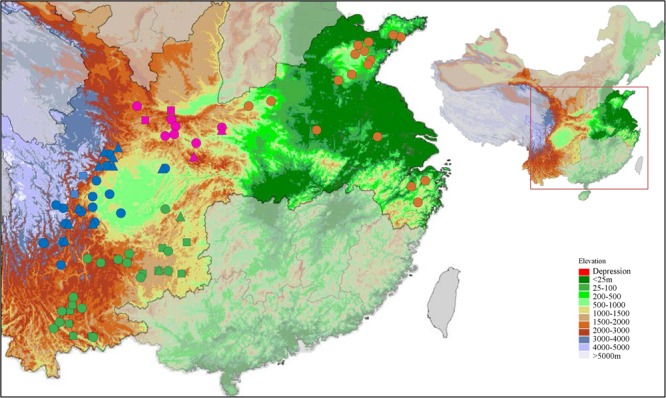
Geographical distributions of 69 Chinese cherry populations and 17 *Cerasus* relative populations. All accessions are collected from natural populations in four geographical regions, including Longmenshan Fault Zones (LFZ, blue), Southwest China (SWC, green), Qinling Mountain (QLM, pink), and North and East China (NEC, orange). The cultivated, wild and *Cerasus* relative populations are colored according to their geographical distributions, and also are represented by circles, triangles, and squares, respectively.

From 2010 to 2014, we investigated and assessed numerous wild and cultivated Chinese cherry accessions distributed in 106 towns of 69 counties in 12 provinces of China (Huang et al., 2013; Chen et al., 2016). After removing both genetically and phenotypically indistinguishable cultivated Chinese cherry accessions, a total of 542 (including 204 wild and 338 cultivated) Chinese cherry accessions were selected from 69 natural populations in four geographical regions including LFZ, SWC, QLM, and North and East China (NEC) (**Figure 1** and **Supplementary Table S1**). About 15 *Cerasus* relative species (162 accessions) were also collected from 15 natural populations to comprehensively estimate the structure pattern and domestication history of Chinese cherry at genus level (**Figure 1** and **Supplementary Table S1**). Two to forty representative accessions were selected within each population (**Supplementary Table S1**). To ensure coverage, the selected accessions in each population were distributed at 50 to 1000 m intervals. The maximum altitude gap between pairwise accessions was 1808 m (WXC) (**Supplementary Table S1**). Our sampling populations have almost covered the whole geographical distributions of Chinese cherry in China. All cultivated Chinese cherry accessions were sampled from local cultivated Chinese cherry resources (not clonally propagated cultivars nor introduced from other places). Among the 338 cultivated Chinese cherry accessions, we grafted 117 accessions from LFZ, SWC, and NEC in a Common Garden in 2013 (Chen et al., 2016). To measure phenotypic traits of fruits and seeds of cultivated Chinese cherries, 86 of 117 grafted cultivated accessions were selected in this study (**Supplementary Table S2**). All grafted cultivated accessions were kept under the same management and growing conditions (Chen et al., 2016).

### Microsatellite Loci and Sample Selection

To reveal the genetic structure and domestication history for cultivated Chinese cherry, we used 20 nuclear microsatellite loci that spread across seven out of the eight linkage groups of the Chinese cherry genome (**Supplementary Table S3**) (Zhang et al., 2016) to genotype a total of 704 accessions (542 Chinese cherry individuals and 162 *Cerasus* relatives). We discarded the locus (L20) with more than 30% missing data, and removed 52 accessions with more than 25% missing data. Finally, 19 loci (L1–L19) and 650 accessions (532 Chinese cherry accessions and 118 *Cerasus* relatives) (**Supplementary Tables S1, S4, S5**) were retained in subsequent analyses.

Because the linked loci and closely related accessions (e.g., fullsibs and parent-offsprings) may lead to spurious genetic structure within populations (Cornille et al., 2012; Yuan et al., 2014), we tested for the pairwise linkage disequilibrium among 19 microsatellite loci in the Genepop software (Rousset, 2008). Relatedness (*r*_xy_) (Lynch and Ritland, 1999) between pairwise accessions within 532 Chinese cherry accessions, or within 326 cultivated Chinese cherry accessions or within 206 wild Chinese cherry accessions were also calculated using the triadic likelihood estimator (TrioML) (Wang, 2007) in the software COANCESTRY (Wang, 2011).

### PCR Amplification and Gel Electrophoresis

Total DNA was extracted from dried leaf tissue with CTAB-based method (Chen et al., 2013). The PCR was performed in a 20-μl reaction volume [1 μl of DNA template (50 ng/μl), 7 μl of ddH_2_O, 0.5 μl each of the forward and reverse primer (10 pmole/μl) and 10 μl of 1 × TIANGEN Taqmix]. The amplification conditions were as follows: 4 min at 94°C, followed by 34 cycles of 50 s at 94°C, 1 min at 53°C, 1.5 min at 72°C, with a final extension of 8 min at 72°C. The PCR products were separated in 8% denaturing polyacrylamide gel. The DNA bands were visualized using the silver staining method (Gao et al., 2009). Band sizes were estimated in Quantity One software (Bio-Rad, United States), via referring to the standard molecular weight size marker (20 bp DNA ladder, TAKARA).

### Genetic Diversity and Bottleneck

We estimated the proportion of null alleles (*P*_n_) with MICROCHECKER 2.2.3 software (Van Oosterhout et al., 2004). The allelic richness (*A*_r_) and private allele richness (*A*_p_) were calculated using a rarefaction approach (Hurlbert, 1971; Kalinowski, 2004) implemented in the ADZE software (Szpiech et al., 2008). The number of alleles (*A*), gene diversity (*H*_s_), observed heterozygosity (*H*_o_) and inbreeding coefficient (*F*_is_) were estimated in FSTAT v2.9.3.2 (Goudet, 2001). Polymorphism information content (*PIC*) for each locus was estimated in POWERMARKER v3.25 software (Van Oosterhout et al., 2004). The Kruskal–Wallis tests on allelic richness (*A*_r_) and gene diversity (*H*_s_) were conducted to investigate whether there were statistical differences in the levels of genetic diversity.

We employed BOTTLENECKE v1.2.02 (Cornuet and Luikart, 1996; Piry et al., 1999) to test for the footprints of genetic bottleneck in Chinese cherry using a Two-phase model (TPM) that allows multiple-step mutations of the Infinite Allele Model (IAM) and Stepwise Mutation Model (SMM) (Luikart et al., 1998). The computation values were set as 10% SMM and 90% IAM with 1000 iterations. Meantime, the occurrence of a genetic bottleneck was also estimated using Garza–Williamson index, which can correctly detect the genetic bottlenecks that just lasted for several generations (Williamson-Natesan, 2005).

### Population Structure and Migration

We used the model-based Bayesian clustering method to infer the population structure in STRUCTURE v2.3.3 (Pritchard et al., 2000). The analyses were performed under six different datasets: (1) full dataset (Chinese cherry accessions and *Cerasus* relatives, N = 650); (2) Chinese cherry dataset (Chinese cherry accessions, *N* = 532); (3) cultivated dataset (cultivated Chinese cherry accessions, *N* = 326); (4) wild dataset (wild Chinese cherry accessions, *N* = 206); (5) less-related cultivated dataset [cultivated Chinese cherry accessions with low relatedness (*r*_xy_ ≤ 0.500), *N* = 69]; (6) pruned dataset [Chinese cherry accessions with Q (membership coefficients) ≥ 0.800, *N* = 344] (**Supplementary Note S1**). Most of the analyses were carried out without the prior information, except for that under wild dataset. Due to the weak signal of structure within wild populations, an extra analysis was performed using geographic sampling locations as prior information to assist clustering of wild populations. For all analyses, *K* ranged from 1 to 12. Twenty independent runs were carried out for each *K*, with the admixture model, correlated allele frequency model, a burn-in of 100,000 and 100,000 subsequent Markov chain Monte Carlo (MCMC) interactions. The most likely number of genetic cluster (*K*) was calculated with the method of Evanno et al. (2005) implemented in the online program STRUCTURE HARVERSTER^1^ (Earl and VonHoldt, 2012). CLUMPP v1.1.2 (Jakobsson and Rosenberg, 2007) was used to look for distinct modes among 20 runs of each *K*. We assigned accessions into a distinct genetic cluster with Q (membership coefficient) ≥ 0.800 and took accessions with intermediate *Q*-values (0.200 < Q < 0.800) as “admixed accessions.”

A principal coordinate analysis (PCoA) was performed with covariance-standardized method in GenAlEx v6.501 (Peakall and Smouse, 2012). Taking *Cerasus* relatives as out group, a rooted NJ (Neighbor-joining) tree was constructed based on *D*_A_ distance (Nei and Chesser, 1983) in the POWERMARKER v3.25 software. The FigTree v1.4.2 software was used to edit and output the final trees.

We also investigated the direction and magnitude of migration among geographical regions and among genetic clusters. Migration networks were generated based on the Nm method (Alcala et al., 2014), using the *divMigrate* function of the R-package (R Development Core Team, 2012) *diveRsity* (Keenan et al., 2013; Sundqvist et al., 2013).

### Demographic Modeling

DIYABC v2.1.0 software (Cornuet et al., 2014) was used to compare different domestication history models and estimate historical parameters in this study. The Generalized Stepwise Mutation model (GSM) was chosen in DIYABC v2.1.0 for microsatellite data. There was no experimental estimation of the mean mutation rate (μ) for Chinese cherry. Referring to the values used in the perennial woody trees (Cornille et al., 2012; Diez et al., 2015), the mean mutation rate was drawn from the uniform distribution with the default values of 10^-4^ and 10^-3^. The mean coefficient *P* and mean *SIN* rate ranged from 0.1 to 0.3 and from 1 0^-8^ to 10^-5^, respectively. Most of the microsatellite loci were suitable to the default allelic range value of 40. The mean mutation rate, mean coefficient *P* and *SIN* rate were also used to characterize each locus, and were drawn from Gamma distribution (shape = 2) with their default values. The summary statistics included mean number of alleles, mean genetic diversity (Nei, 1978), genetic differentiation between pairwise groups (*F*_ST_) (Weir and Cockerham, 1984) and genetic distance (δμ)^2^ (Goldstein et al., 1995).

1000,000 simulated datasets were generated for each model for further analyses. We performed a pre-evalution to check whether our model and parameter prior definition were fit for subsequent analyses. The model checking analysis was carried out to evaluate how well the model and priors of parameters fit the data summarized by summary statistics. To compute the posterior probability of each model, a linear discriminant analysis was conducted using the polychotomous logistic regression with 95% confidence. We employed a local linear regression to estimate the posterior distributions of parameters under the best-fit model. The 1% simulated datasets mostly resemble the observed data were used for regression after logit transformation.

### *Q*_ST_–*F*_ST_ Comparison

Seven quantitative traits of fruits and seeds included fruit diameter (cm), fruit *trans*-diameter (cm), fruit shape index, length of carpopodium (cm), stone length (cm), stone width (cm) and stone thickness (cm) (**Supplementary Table S2**). The data of quantitative traits were obtained from “Common-Garden” environment. Boxplots were drawn for seven quantitative traits using the *Box Plots* function in *R* statistical software. The *Q*_ST_–*F*_ST_ comparison method (Lande, 1992; Whitlock, 2008) was used to compare genetic and phenotypic differentiation based on microsatellites and quantitative traits. Neutrality test for microsatellites loci was conducted based on a *F*_ST_-outlier method in LOSITAN (Antao et al., 2008), and outlier loci were removed in each comparison. *Q*_ST_ values for each trait were calculated using the following expression: *Q*_ST_ = *V*_G,among_/(*V*_G,among_ + 2*V*_A,within_), where *V*_A,within_ is the additive genetic variance within populations and *V*_G,among_ is the genetic variance among populations (Lande, 1992; Spitze, 1993). The *F*_ST_ values (Weir and Cockerham, 1984) were also calculated with 10000 bootstraps in *R* according to the methods of Whitlock and Guillaume (2009). We compared *Q*_ST_ and *F*_ST_ values in each case to investigate whether phenotypic divergences can be statistically explained by only genetic drift (*Q*_ST_ = *F*_ST_), divergent selection (*Q*_ST_ > *F*_ST_) or stabilizing selection (*Q*_ST_ < *F*_ST_).

## Results

### Moderate Genetic Diversity and Significant Domestication Bottleneck

In this study, we analyzed a total of 532 Chinese cherry accessions and 118 *Cerasus* relatives from LFZ, SWC, QLM, and NEC that represented their mainly geographical distributions in China (**Figure 1** and **Supplementary Table S1**). At 19 nuclear microsatellite loci, a total of 235 alleles were genotyped across 650 accessions, with an average of 12.4 per locus (**Supplementary Table S4**). Gene diversity (*H*_s_) was between 0.325 and 0.783, with an average of 0.559. Mean polymorphism information content (*PIC*) was 0.523, ranging from 0.320 to 0.757. No or low pairwise linkage disequilibrium was detected in a subset of 69 Chinese cherry populations among 19 microsatellite loci.

Moderate genetic diversity was detected in Chinese cherry at species level (*A*_r_ = 1.464, *H*_s_ = 0.464) (**Table 1**). Cultivated Chinese cherry (*A*_r_ = 1.402, *H*_s_ = 0.402) revealed significantly lower genetic diversity than wild Chinese cherry (*A*_r_ = 1.531, *H*_s_ = 0.532) (all *P* < 0.05; Kruskal–Wallis tests on *A*_r_ and *H*_s_), indicating a moderate loss of genetic diversity in cultivated accessions (**Table 1**). Among different geographical regions, the highest genetic diversity was detected in LFZ-W (*A*_r_ = 1.532, *H*_s_ = 0.533) (**Table 1**). The two lowest levels of genetic diversity were observed in LFZ-C (*A*_r_ = 1.303, *H*_s_ = 0.303) and NEC-C (*A*_r_ = 1.376, *H*_s_ = 0.376). The *F*_is_ value of Chinese cherry was 0.261 (*P* < 0.01) (**Table 1**). High and significant *F*_is_ values were detected in wild Chinese cherry, while low or negative *F*_is_ values were observed in cultivated Chinese cherry (**Table 1** and **Supplementary Table S6**).

**Table 1:** The genetic diversity, inbreeding coefficients, Garza–Williamson index and T.P.M values in Chinese cherry.

Types	Origin	*N*	*A*	*A*_r_	*A*_p_	*H*_s_	*H*_o_	*F*_is_	GW	T.P.M
**Species level**		532	6.7	1.464	–	0.464	0.343	0.261^∗∗^	0.247	0.000^∗∗∗^
**Putative status**									
	Cultivated	326	5.2	1.402^a^	0.508	0.402^a^	0.354	0.121^∗∗^	0.252	0.000^∗∗∗^
	Wild	206	6.4	1.531^b^	0.637	0.532^b^	0.324	0.391^∗∗^	0.253	0.000^∗∗∗^
**Geographical regions**									
	LFZ-C	109	4.1	1.303^a^	0.082	0.303^a^	0.337	–0.100^ns^	0.261	0.000^∗∗∗^
	SWC-C	82	3.8	1.398^ab^	0.115	0.398^abc^	0.348	0.126^∗∗^	0.246	0.016^∗∗^
	QLM-C	30	3.2	1.411^ab^	0.106	0.412^abcd^	0.367	0.110^∗∗^	0.269	0.490^ns^
	NEC-C	105	3.8	1.376^a^	0.099	0.376^ab^	0.375	0.001^ns^	0.258	0.104^ns^
	WLFZ-W	170	6.2	1.532^c^	0.243	0.533^d^	0.331	0.378^∗∗^	0.251	0.000^∗∗∗^
	SWC**-**W	17	3.9	1.524^c^	0.259	0.534^d^	0.277	0.482^∗∗^	0.257	0.145^ns^
	QLM**-**W	19	3.8	1.479^bc^	0.182	0.485^bcd^	0.284	0.415^∗∗^	0.250	0.060^ns^

*N, sample size; A: the number of alleles; A_*r*_, the allelic richness averaged across loci estimated by rarefaction using a standard sample size of two; A_*p*_, private allele richness averaged across loci estimated by rarefaction using a standard sample size of two; H_*s*_, gene diversity; H_*o*_, observed heterozygosity; F_*is*_, inbreeding coefficient; GW, Garza–Williamson index; T.P.M, two-phased model of mutation; ^∗^P < 0.05, ^∗∗^P < 0.01, ^∗∗∗^P < 0.001; ns, not significant. The lower-case letters by the A_*r*_ and H_*s*_ values represent significance groupings after a pairwise comparison (P < 0.05; Kruskal–Wallis test) between putative status, and between geographical regions.*

The footprints of genetic bottleneck were comprehensively estimated in Chinese cherry. BOTTLENECK analyses indicated significant evidence of a bottleneck in cultivated Chinese cherry, LFZ-C, SWC-C and numerous cultivated populations (**Table 1** and **Supplementary Table S6**). Garza–Williamson indices were lower than the threshold value of 0.68 in all populations and in all geographical regions (**Table 1** and **Supplementary Table S6**). Overall, our results suggested that cultivated Chinese cherry experienced significant genetic bottlenecks during domestication.

### Genetic Clustering and Migration Networks

We calculated the pairwise relatedness values (*r*_xy_) between Chinese cherry accessions before STRUCTURE analysis. The percentage of pairs with *r*_xy_ over 0.500 was as follows: 5.219% in 532 Chinese cherry accessions (*N* = 7372), 0.298% in 206 wild Chinese cherry accessions (*N* = 63), and 13.167% in 326 cultivated Chinese cherry accessions (*N* = 6975). Given the high proportion of correlated accessions in cultivated Chinese cherry, a structure analysis was carried out using less-related cultivated dataset (*N* = 69). The analysis obtained similar structure patterns to those under cultivated dataset (*N* = 326) (**Supplementary Figure S1**). Therefore, we retained all cultivated Chinese cherry accessions for subsequent structure analyses.

**FIGURE 2:**
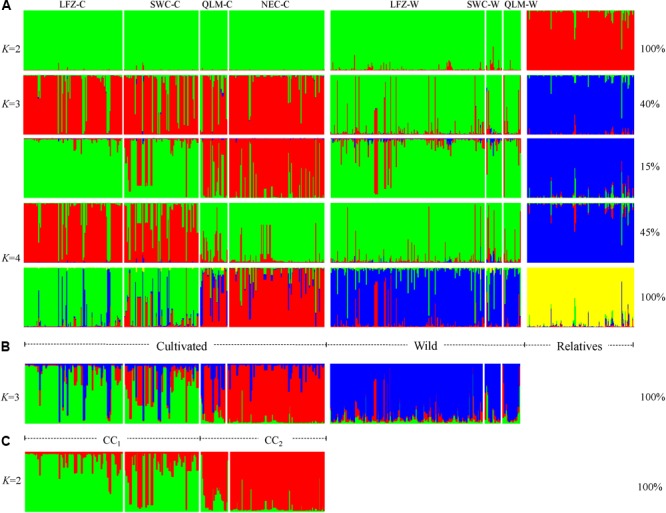
The results of STRUCTURE analyses under **(A)** full dataset (*N* = 650), **(B)** Chinese cherry dataset (*N* = 532), and **(C)** cultivated dataset (*N* = 326). A vertical line represents an individual. The *x*-axis shows different populations, putative status of the samples (cultivated, wild or *Cerasus* relative), and genetic clusters. The proportion of each simulation represents by each mode is shown.

Model-based Bayesian clustering method was used to infer the population structure and gene introgression of Chinese cherry with different datasets in our STRUCTURE analyses. According to the method of Evanno et al. (2005), the greatest Delta *K* was detected at *K* = 2 under full dataset (*N* = 650) (**Supplementary Figure S2A**), where Chinese cherry and its *Cerasus* relatives showed two distinct genetic patterns with little gene introgression between each other (**Figure 2A**). Three genetic patterns and recent gene introgression were found under Chinese cherry dataset (*N* = 532) at the optimal Delta *K* (*K* = 3) (**Figure 2B, Supplementary Figure S2B**, and **Supplementary Table S7**). Wild Chinese cherry showed one separate structure pattern (**Figure 2B** and **Supplementary Figure S3**). Cultivated Chinese cherry exhibited rough geographical structure patterns (**Figure 2B**) that could also be identified by our analysis under cultivated dataset (**Figure 2C** and **Supplementary Figure S2C**).

**FIGURE 3:**
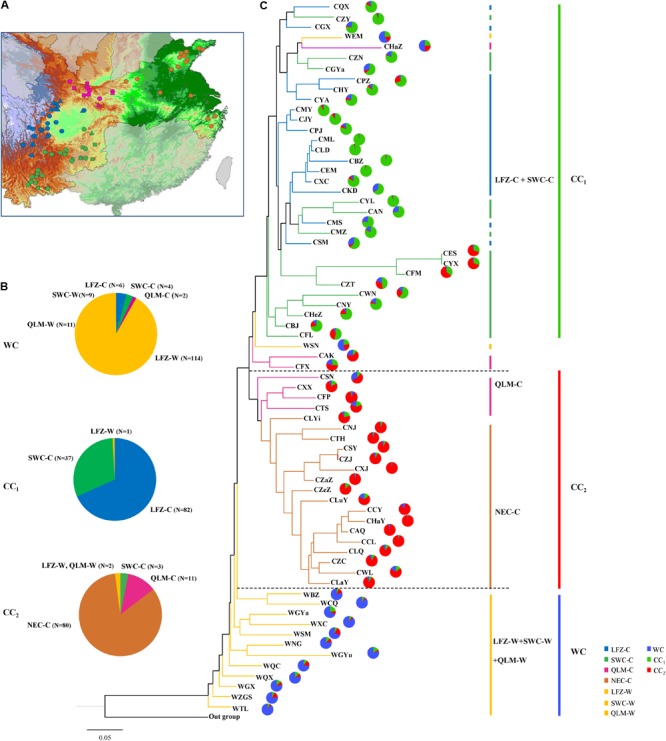
The clustering results of Chinese cherry based on the Bayesian clustering approach and neighbor-joining (NJ) analysis. **(A)** Sampling population location in four geographical regions. The symbol and color of each population corresponds to those in **Figure 1**. **(B)** Compositions of three genetic clusters (WC, CC_1_, and CC_2_). **(C)** Rooted NJ tree constructed based on *D*_A_ distance and genetic structure patterns of 69 Chinese cherry populations identified by STRUCTURE analysis under the Chinese cherry dataset (*N* = 532). In **(B,C)**, the colors of LFZ-C, SWC-C, QLM-C, and NEC-C populations correspond to those of **(A)**, whereas LFZ-W, SWC-W, and QLM-W populations are uniformly colored by yellow.

We defined one wild (WC) and two cultivated genetic clusters (CC_1_ and CC_2_) in Chinese cherry according to the STRUCTURE analysis under Chinese cherry dataset with Q ≥ 0.800 (**Supplementary Table S8**). At *K* = 3, 134 wild accessions and 12 cultivated accessions were grouped into WC (**Supplementary Table S8** and **Figures 3A,B**). The assignment of cultivated Chinese cherry accessions showed significant geographical signals (**Figures 3A,B** and **Supplementary Table S8**). CC_1_ was mainly composed of 82 LFZ-C accessions and 37 SWC-C accessions, while CC_2_ was mainly consisted of 11 QLM-C accessions and 80 NEC-C accessions (**Figure 3B** and **Supplementary Table S8**). The genetic clustering in Chinese cherry was further investigated with the neighbor-joining (NJ) analysis and PCoA. In the NJ tree, all LFZ-C and SWC-C populations were clustered into one clade, while four QLM-C populations and all NEC-C populations were grouped into another, corresponding to their genetic patterns identified in STRUCTURE analyses (**Figure 3C**). PCoA analyses revealed similar clustering results to those in STRUCTURE and NJ analyses (**Supplementary Figures S4A,B**). Moreover, our PCoA showed distinct genetic compositions between CC_1_ and CC_2_ (**Supplementary Figure S4C**), suggesting their differentiated genetic compositions.

**FIGURE 4:**
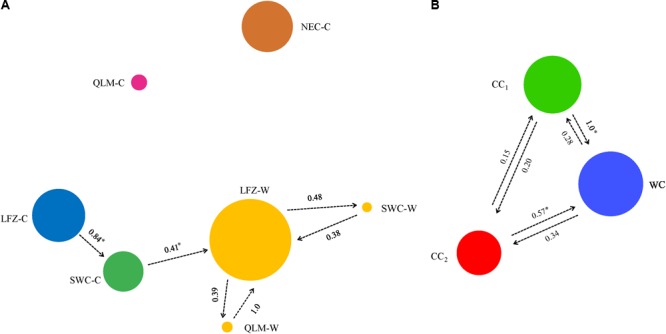
Relative *divMigrate* networks for **(A)** Chinese cherry among different geographical regions and **(B)** three genetic clusters identified by STRUCTURE analysis using the *divMigrate* function in *diveRsity*. For **(A,B)**, the size of the circle illustrates the population size, and the colors are coincident with those in **Figure 3B**. The asymmetric values are shown alongside each arrow. In A, we only show the pairwise relationships with asymmetric values over 0.35. Both networks are generated following 1,000 bootstraps. Among all pairwise relationships, only those from LFZ-C to SWC-C, from SWC-C to LFZ-W, from CC_1_ to WC and from CC_2_ to WC are significant. ^∗^*P* < 0.05.

We constructed the relative *divMigrate* networks with Nm method to assess the migration patterns and gene flow of Chinese cherry (*C. pseudocerasus*) among different geographical regions, and among different genetic clusters defined by STRUCTURE (**Figures 4A,B**). In the networks, QLM-C and NEC-C showed low migration (all asymmetric values < 0.35) with LFZ-C and SWC-C (**Figure 4A**), suggesting a relative separate domestication process among these regions. Interestingly, we found no considerable migration from wild to cultivated Chinese cherry (all asymmetric values < 0.35) but detected moderate migration from SWC-C to LFZ-W (**Figure 4A**). Among the three genetic clusters, the highest migration was observed from CC_1_ to WC, and the lowest was detected from CC_2_ to CC_1_ (**Figure 4B**).

In summary, STRUCTURE analyses found no contributions from *Cerasus* relatives (RC) to the evolution of Chinese cherry, and showed one separate structure in wild Chinese cherry and two rough geographical structures in the cultivated cherry. STRUCTURE, NJ and PCoA analyses all assigned Chinese cherry accessions into three genetic clusters (WC, CC_1_, and CC_2_). Recent gene introgression was only observed within Chinese cherry, yet no considerable migration was detected from wild to cultivated Chinese cherry and among three genetic clusters in our *divMigrate* networks. Since *Cerasus* relatives (RC) were highly diverged from Chinese cherry, we did not include them in our further analyses.

### Phenotypic Traits Associated with Significant Domestication

To investigate whether the phenotypic differentiation of fruit and seed traits are significantly associated with the domestication, seven quantitative traits were measured in 86 grafted cultivated Chinese cherry accessions in this study (**Supplementary Table S2**). Boxplots showed that most of quantitative traits show significant differences among geographical regions, and between two cultivated genetic clusters (CC_1_ and CC_2_) (**Supplementary Figure S5**). Only *Q*_ST_ values of fruit shape index and length of carpopodium were significantly higher than the average *F*_ST_ values among different geographical regions (**Table 2**), suggesting divergent selection during domestication in the two traits. Between cultivated genetic clusters, significant divergent selection was only detected in the fruit shape index (**Table 2**). For all seed traits, no *Q*_ST_ values were significantly different from the *F*_ST_ values either among geographical regions, or between CC_1_ and CC_2_ (**Table 2**).

**Table 2:** *Q*_ST_ values for each trait and the mean *F*_ST_ values between genetic clusters and among geographies.

Phenotypic traits	Between genetic clusters		Among geographical regions	
	*Q*_ST_ values	*F*_ST_ values	*Q*_ST_ values	*F*_ST_ values
Fruit diameter	0.145	0.099	0.133	0.081
Fruit *trans*.diameter	0.006	[0.001, 0.227]	0.025	[0.020, 0.150]
Fruit shape index	0.313		0.257	
Length of carpopodium	0.223		0.199	
Stone length	0.021		0.031	
Stone width	0.090		0.075	
Stone thickness	0.077		0.024	

*The F_*ST*_ values are estimated with the median and boundaries of the 95% credibility intervals (CI2.5 and CI97.5) with 10000 bootstraps.*

### Inference of Domestication History

**FIGURE 5:**
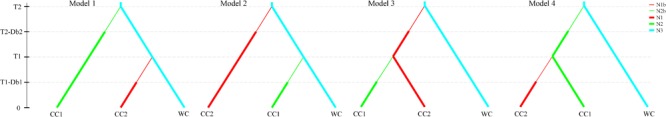
Four Chinese cherry domestication models compared in approximate Bayesian computation (ABC) analysis. In model 1, we assume that CC_1_ is first split from WC, and then CC_2_ is separated from WC. We also set an independent domestication history for CC_1_ and CC_2_, but assume that CC_2_ is derived from WC first in model 2. For model 3, we assume that CC_2_ is derived from WC first, and subsequently CC_1_ is derived from CC_2_. In model 4, we assume that CC_1_ is derived from WC first, and then CC_2_ diverges from CC_1_. We design domestication bottlenecks for both CC_1_ and CC_2_ in each model. T1: the divergent time of the second domestication event; T2: the divergent time of the first domestication event; Db1 and Db2: duration of the bottlenecks. N1: effective size of CC_1_; N2: effective size of CC_2_; N3: effective size of WC; N1b: effective size of CC_1_ at the time of domestication; N2b: effective size of CC_2_ at the time of domestication

The ABC analysis was conducted using pruned data (*N* = 344) (**Supplementary Note S1**) to infer the domestication history of cultivated Chinese cherry. According to historical records, we assumed that no significant admixture events had occurred among different genetic clusters after the original domestication event(s). Four simple models were designed for Chinese cherry (**Figure 5**). Since the earliest fossil evidence and written records indicated that the domestication of Chinese cherry occurred over 3000 ya (years ago) (Liu et al., 2008), we set a uniform prior distribution for T2 [697, 1700] (**Supplementary Table S9**).

The relative posterior probabilities of four models were estimated to find the best-fit model for Chinese cherry. Moderate probabilities were detected in the four models, with a range between 0.155 (model4) and 0.376 (model2). We detected relatively higher probabilities for two independent domestication history models (0.659) than two dependent models (0.341) (**Supplementary Table S10**). To estimate whether the superiority is stable for independent domestication history models, we also carried out the other five comparing analyses between independent and dependent domestication models. All of the probabilities in models 1 and 2 (0.6189–0.6953) were much higher than those in models 3 and 4 (0.3047–0.3811) (**Supplementary Table S11**). Between the two independent domestication models, higher probabilities were detected for model 2 than that for model 1 (**Supplementary Tables S10, S11**). Therefore, we took the model 2 as the best-fit domestication model for Chinese cherry. The type I error and mean type II error for model 2 were 0.120 and 0.051, respectively, showing a good confidence for our best model choices.

The posterior distributions of parameters were estimated only for model 2 (**Figure 5**). Because the juvenile period of Chinese cherry lasts three to 6 years, we assumed a mean generation time of 4.5 yr (year). In model 2, CC_2_ was split from WC 3924 ya (95% CI: [3334.5, 7020]), and then CC_1_ was divergent from WC 2237 ya (95% CI: [715.5, 3798]) (**Table 3**). We detected long-lasting original domestication bottlenecks in CC_1_ and CC_2_. The durations of the bottlenecks were 1598 yr in CC_1_ and 3938 yr in CC_2_, and the current effective population sizes of CC_1_ (N1 = 1500, 95% CI: [257, 3170]) and CC_2_ (N2 = 1060, 95% CI: [108, 3030]) were much smaller than that of WC (N3 = 6730, 95% CI: [3840, 9580]) (**Table 3**).

**Table 3:** The parameter posterior distributions of model 2 under pruned dataset (*N* = 344) in approximate Bayesian Computation analysis.

Parameter	Pruned dataset (*N* = 344)		
	Median	CI2.5	CI97.5
N1(CC_1_)	1500	257	3170
N2(CC_2_)	1060	108	3030
N3(WC)	6730	3840	9580
N1b	504	325	2880
N2b	1270	288	3080
T1	497	159	844
T2	872	741	1560
Db1	355	18.6	841
Db2	875	48.6	1660
μ	1.14e-04	1.00e-04	1.87e-04
P	2.57e-01	1.32e-01	3.00e-01
μSNI	1.89e-07	1.11e-08	6.56e-06

*N1, effective size of CC_*1*_; N2, effective size of CC_*2*_; N3, effective size of WC; N1b, effective size of CC_*1*_ at the time of domestication; N2b, effective size of CC_*2*_ at the time of domestication; T1, the divergent time of the second domestication event; T2, the divergent time of the first domestication event; Db1 and Db2, duration of the bottleneck; μ, mean nucleotide mutation rate; P, mean coefficient P; μSNI, mean single nucleotide indel mutation rate. All posterior distributions are estimated with the median and boundaries of the 95% credibility intervals (CI2.5 and CI97.5).*

We also compared the four domestication models with the Chinese cherry dataset (*N* = 532). According to the results of genetic clustering and migration, we assigned LFZ-C and SWC-C into one group (cultivated group 1, CG_1_) and clustered QLM-C and NEC-C into another one (cultivated group 2, CG_2_) without removing the “admixed accessions.” The ABC analysis obtained similar results to those using the pruned dataset (**Supplementary Tables S10–S12**). Overall, our ABC analyses based on different datasets identically revealed an independent domestication history and long-lasting domestication bottlenecks in cultivated Chinese cherry.

## Discussion

Chinese cherry is an important perennial woody fruit crop with self-compatible system. In this study, we comprehensively investigated the genetic structure and domestication history of Chinese cherry. Overall, a separate genetic structure was detected in wild Chinese cherries and rough geographical structures were observed in cultivated Chinese cherries in our STRUCTURE analyses. One wild (WC) and two cultivated (CC_1_ and CC_2_) genetic clusters were defined in Chinese cherry according to STRUCTURE, Neighbor-Joining and PCoA analyses. We provide robust molecular evidence for the multiple origins and independent domestication histories in cultivated Chinese cherry for the first time. Little genetic contributions from *Cerasus* relatives (RC) were detected in the evolution and domestication of Chinese cherry. Also, frequent clonal propagation probably leads to moderate loss of genetic diversity and long-lasting domestication bottlenecks in cultivated Chinese cherry. All of these results are markedly different from the strictly out-crossing perennial woody fruit crop, domesticated apples (Cornille et al., 2012, 2014; Duan et al., 2017).

### Moderate Loss of Genetic Diversity and Long-Lasting Genetic Bottlenecks in Cultivated Chinese Cherry

Domestication is a human-mediated evolutionary process and impacts contemporary patterns of genetic variation in domesticated populations. Genetic bottlenecks are expected to occur during domestication of plant species (Gross and Olsen, 2010). However, life-history traits specific to trees and outcrossing system can reduce the occurrence of domestication bottlenecks in fruit trees. For example, as a strictly outcrossing perennial woody fruit crop, domesticated apples maintain high levels of genetic diversity, and have not undergone genetic bottlenecks during domestication (Cornille et al., 2012, 2014; Duan et al., 2017). Different from domesticated apples, our results showed that cultivated Chinese cherry experienced moderate loss of genetic diversity and over 1000-year genetic bottlenecks during domestication. Chinese cherry is a rare self-compatible perennial woody fruit crop in the Rosaceae family. During long-term cultivation history, Chinese cherries were both sexually (seedlings) and clonally (suckers or cuttings) propagated. Rooting sucker and scion grafting are the foremost modes of clonal reproduction for cultivated Chinese cherries. Therefore, we consider that the frequent rooting sucker and scion grafting during cultivation history retain desirable cultivated accessions with same or similar genetic patterns, thereby leading to the moderate loss of genetic diversity and long-lasting genetic bottlenecks in cultivated Chinese cherry. The low and negative *F*_is_ values in cultivated Chinese cherries also support our viewpoint, since frequent clonal propagation can lead to the heterozygote excess in domesticated plants (McKey et al., 2010).

### Independent Domestication History and Potential Original Domestication Center

The understanding of plant domestication can reveal the history of human civilization and guide modern breeding programs (Miller and Gross, 2011). Core questions about plant domestication mainly include number of domestication event(s), the divergent time between domesticated plant and its wild ancestors, and domestication center, all of which have not been answered in reported researches of Chinese cherry. Here, our study probed deeply and thoroughly into these questions based on our molecular and phenotypic data.

Our ABC analyses selected model 2 as the best-fit domestication model for cultivated Chinese cherry (**Figure 5**). We have not detected considerable gene introgression and migration from wild to cultivated Chinese cherry, and between cultivated genetic clusters (CC_1_ and CC_2_). Comparing to the ABC approach, the STRUCTURE analyses trend to reveal the recent dynamic patterns among different populations or clusters (Pritchard et al., 2000; Anderson and Thompson, 2002; Excoffier et al., 2005). Therefore, we ignore the gene introgression and migration among different genetic clusters while designing the domestication models. Moderate posterior probabilities were detected in model 2. Posterior probabilities can be influenced by the combination of models included in an ABC analysis (Pelletier and Carstens, 2014). In this study, we obtain four highest posterior probabilities (0.6189–0.6953) in two independent domestication models (models 1 and 2) among all pairwise comparing models (**Supplementary Table S11**). Therefore, we consider that the moderate posterior probabilities in model 2 result from its high similarity with model 1 in our modeling designs.

In model 2, the ABC estimations provide strong supports for the independent domestications for cultivated Chinese cherries, which can also be supported by the genetic clustering analyses, *divMigrate* networks and the *Q*_ST_–*F*_ST_ comparison. The lowest genetic diversity has been detected in LFZ-C and NEC-C among four geographical regions. Therefore, LFZ and NEC are the two most possible major domestication sites of cultivated Chinese cherries, where cultivated accessions experienced divergent selections in fruit traits caused by different domestication trends and preferences.

Our study provides a deep insight in the number of domestication events and the divergent times between wild and cultivated Chinese cherries. The ABC estimations detected multiple origins with two independent domestication events in cultivated Chinese cherries. The first domestication event occurred about 3900 ya and the second one took place about 2200 ya, which correspond to the Xia dynasty (2070-1600 BC) and the Spring and Autumn Period (770-221 BC), respectively. Long-lived perennial trees are thought to be domesticated later than annuals (Miller and Gross, 2011). As expected, two domestication events in cultivated Chinese cherries happened around the same time to those of other perennial woody fruit crops in the Rosaceae family, such as apple (Zohary and Spiegel-Roy, 1975; Zohary and Hopf, 1994) and peach (Zheng et al., 2014), but occurred much later than those of rice (Vaughan et al., 2008), and soybean (Hymowitz, 1970). One hypothesis proposes that tree domestication have not emerged until people could clone trees through clonal reproduction (Zohary and Spiegel-Roy, 1975). In our data, the first domestication event occurred around the time when the perennial woody fruit trees can easily be clonally propagated with simple techniques (e.g., cuttings and suckers). The second one happened accompanying the wide dispersal of scion grafting between 3000 and 2000 ya. Our results indeed provide strong molecular evidences for the high correlation between the domestication events and clonal propagation techniques in perennial woody fruit crops. Moreover, the occurrences of the two independent domestication events in cultivated Chinese cherries are also closely linked to the spread of agricultural civilization and trade. Ancient civilization of mankind is actually a farming civilization, representing the agricultural civilization and trade to a certain extent. In the ancient China, NEC has been the cultural, political and agricultural center since the Xia dynasty (2070-1600 BC), when the agricultural activities were extremely stagnant in the other regions. Until the Spring and Autumn Period (770-221 BC), the cultivation technologies and trade in LFZ have been drastically promoted accompanying with the population increase and the expansion of agricultural areas.

It is important to confirm the original center of the domestication of cultivated Chinese cherries for revealing their potential dispersal routes. In our data, we detected the highest genetic diversity in LFZ-W among all regions (**Table 1** and **Supplementary Table S6**). Abundant wild Chinese cherry populations, and phenotypically distinguishable wild types corresponding to the common cultivated types, have been found only in LFZ. Combined with our phylogeographic data using chloroplast and nuclear DNA sequences (Chen et al., 2014), LFZ is the most likely region of the domestication center of all cultivated Chinese cherries. Thus, after the first domestication event in LFZ, cultivated Chinese cherries (CC_2_) spread to NEC along the gallery roads of QLM. Subsequently, other cultivated Chinese cherries (CC_1_) were domesticated throughout LFZ (the second event) and dispersed to SWC through the ancient tea horse road.

## Conclusion

In this study, we obtained an overview of the genetic structure and domestication history of Chinese cherry at species and genus level. A separate genetic structure was detected in wild Chinese cherries and rough geographical structures were observed in cultivated Chinese cherries. Further, our study is the first to reveal the duration of domestication bottlenecks and divergent times between wild and cultivated Chinese cherries. Two domestication events have been found in Chinese cherries. LFZ has been inferred to be the potential original center of the domestication for cultivated Chinese cherries. Overall, our study showed a rare example of a perennial woody fruit crop with multiple origins and independent domestication history, and provided important references for revealing the demographic history of perennial woody fruit trees.

## Data Accessibility

Sampling locations, morphological data and microsatellite genotypes have been listed in our **Supplementary Tables S1–S3**.

## Author Contributions

JZ designed this research, performed the experiments, analyzed the data, and wrote the manuscript. XW designed this research and revised the manuscript. TC and HT designed the partial research and revised the manuscript. YW and QC revised the manuscript. BS, YL, and YZ contributed the materials or analytical tools.

## Conflict of Interest Statement

The authors declare that the research was conducted in the absence of any commercial or financial relationships that could be construed as a potential conflict of interest.

## Footnotes

1 http://taylor0.biology.ucla.edu/structureHarvester/

## Supplementary Material

FIGURE S1 The Bayesian clustering results under **(A)** cultivated dataset (*N* = 326) and **(B)** less-related cultivated Chinese cherry dataset (*N* = 69). Both of the results show two similar genetic clusters in cultivated Chinese cherry accessions, indicating that correlated cultivated accessions have limited effects on the estimation of genetic structure within cultivated accessions.

FIGURE S2 The most likely numbers (Δ*K*) of genetic clusters under **(A)** full dataset (*N* = 650), **(B)** Chinese cherry dataset (*N* = 532), and **(C)** cultivated dataset (*N* = 326).

FIGURE S3 Genetic structure among wild Chinese cherry populations **(A)** without prior information and **(B)** with sampling locations as prior information.

FIGURE S4 The first two axes of a principal component analysis (PCoA) representing the microsatellite data. **(A)** Clustering results of 650 Chinese cherry accessions and their *Cerasus* relatives. *Cerasus* relatives (RC) are marked with gray circles. Chinese cherry accessions from different geographical regions are represented by different symbols and colors. Similar to STRUCTURE, distinct genetic patterns are observed between Chinese cherry accession and its *Cerasus* relatives. **(B)** The assignments of 532 Chinese cherry accessions. The symbols and colors for each Chinese cherry accession are in agreement with those of **(A)**. Clustering results show weak geographical signal in cultivated Chinese cherry. **(C)** Principle coordinate analysis of Chinese cherry accessions in WC (deep blue), CC_1_ (green), and CC_2_ (red). CC_1_ and CC_2_ are distributed in two different sides of WC without any overlapping, which indicates the separate genetic compositions between them.

FIGURE S5 Box plots of seven quantitative traits of cultivated Chinese cherry accessions **(A)** between different geographical regions and **(B)** between two cultivated genetic clusters.

TABLE S1 The summary of taxonomic position, geographical regions, coordinates, sample size of selecting (*N* = 704) and finally used (*N* = 650) Chinese cherry and *Cerasus* relative species.

TABLE S2 Fourteen qualitative and quantitative traits measured and calculated for grafted cultivated accessions.

TABLE S3 Repeat motifs, primer sequences, annealing temperature and linkage groups of 20 microsatellite loci in this study.

TABLE S4 Genetic characteristics of finally used 19 microsatellite loci across 650 accessions.

TABLE S5 Raw molecular data of 650 accessions at 19 nuclear microsatellite markers.

TABLE S6 Genetic polymorphism, inbreeding coefficients and bottleneck of 69 Chinese cherry populations.

TABLE S7 Mean proportions of assignments at *K* = 3 between wild and cultivated Chinese cherry under Chinese cherry (*N* = 532) and pruned dataset (*N* = 344).

TABLE S8 Membership coefficients (*Q*-values) at *K* = 3 inferred from the STRUCTURE analysis under Chinese cherry dataset (*N* = 532).

TABLE S9 Uniform prior information for four domestication models in DIYABC.

TABLE S10 The probabilities of four models under pruned (*N* = 344) and Chinese cherry (*N* = 532) datasets in approximate Bayesian computation (ABC) analyses.

TABLE S11 The probabilities competing between dependent and independent models, and between models 1 and 2 under pruned (*N* = 344) and Chinese cherry (*N* = 532) dataset.

TABLE S12 The Demographic and mutation parameters estimated using approximate Bayesian computation (ABC) for model 2 under Chinese cherry dataset (*N* = 532).

NOTE S1 The details on the assignments of Chinese cherry accessions in the pruned dataset (*N* = 344).
