# Supplementary figures and images for: Genetic Diversity and Domestication Footprints of Chinese Cherry [Cerasus pseudocerasus (Lindl.) G.Don] as Revealed by Nuclear Microsatellites
Source: Front Plant Sci. 2018 Feb 27;9:238. doi: 10.3389/fpls.2018.00238 (PMC5835088; doi:10.3389/fpls.2018.00238)

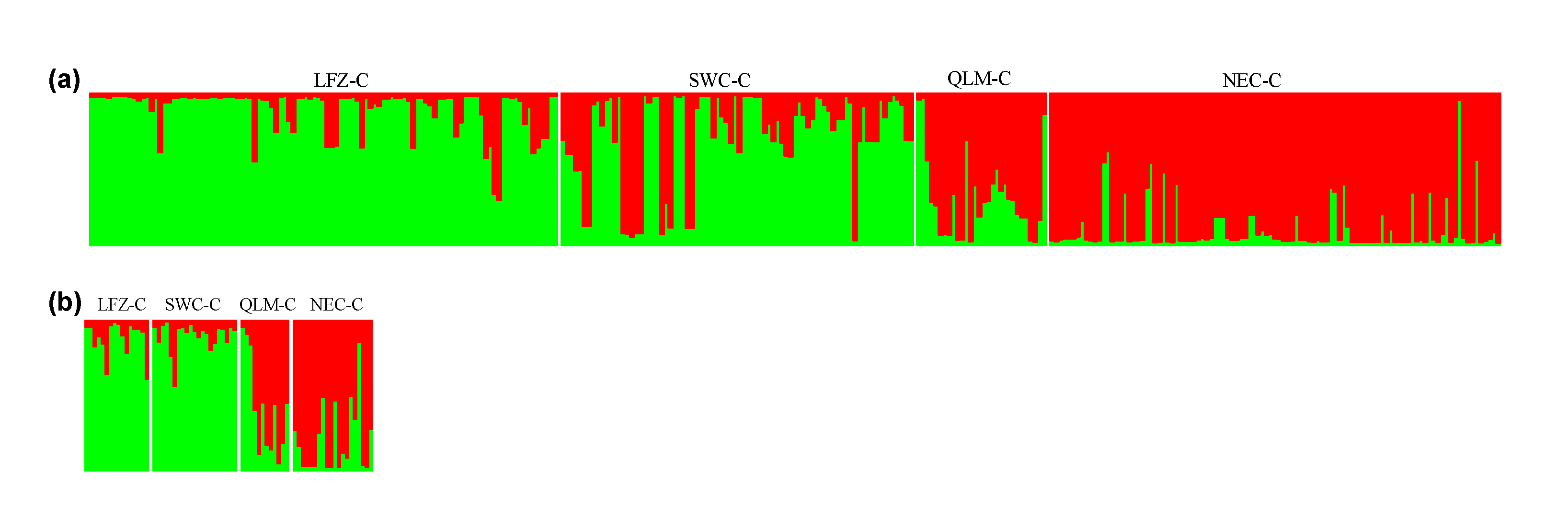

Supplement: FIGURE S1 — The Bayesian clustering results under (A) cultivated dataset (N = 326) and (B) less-related cultivated Chinese cherry dataset (N = 69). Both of the results show two similar genetic clusters in cultivated Chinese cherry accessions, indicating that correlated cultivated accessions have limited effects on the estimation of genetic structure within cultivated accessions. [file Image_1.TIFF]

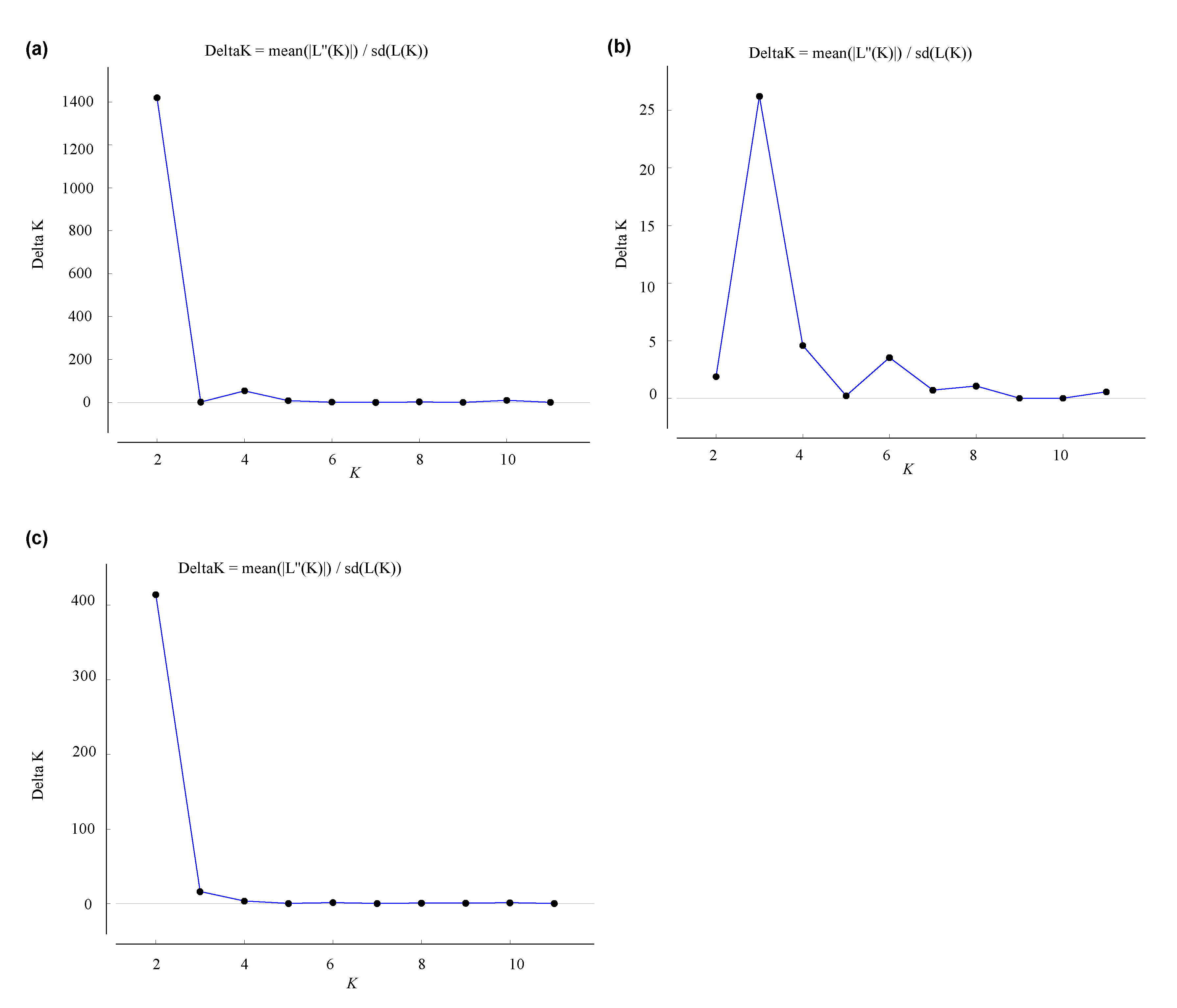

Supplement: FIGURE S2 — The most likely numbers (ΔK) of genetic clusters under (A) full dataset (N = 650), (B) Chinese cherry dataset (N = 532), and (C) cultivated dataset (N = 326). [file Image_2.TIFF]

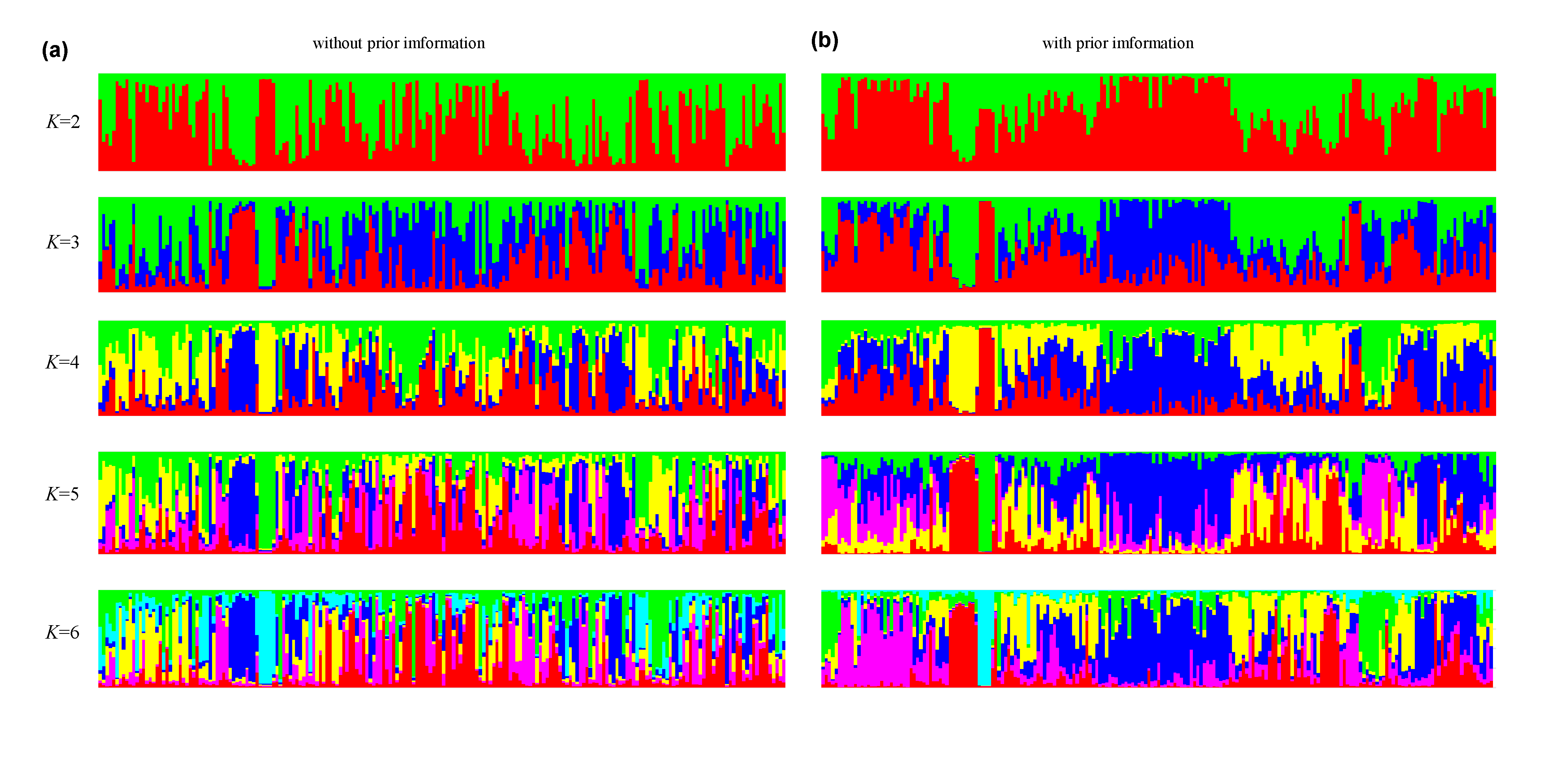

Supplement: FIGURE S3 — Genetic structure among wild Chinese cherry populations (A) without prior information and (B) with sampling locations as prior information. [file Image_3.TIFF]

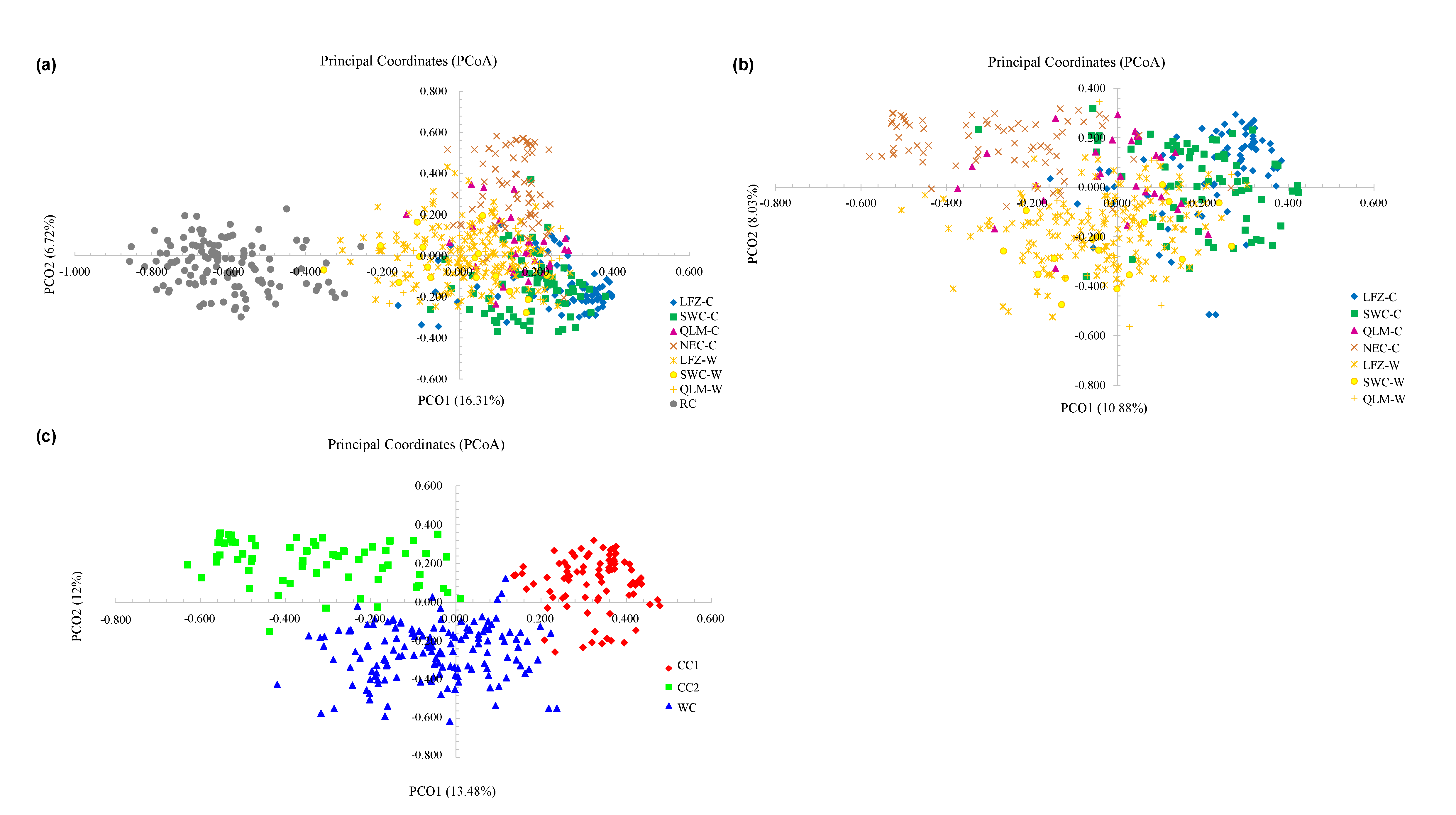

Supplement: FIGURE S4 — The first two axes of a principal component analysis (PCoA) representing the microsatellite data. (A) Clustering results of 650 Chinese cherry accessions and their Cerasus relatives. Cerasus relatives (RC) are marked with gray circles. Chinese cherry accessions from different geographical regions are represented by different symbols and colors. Similar to STRUCTURE, distinct genetic patterns are observed between Chinese cherry accession and its Cerasus relatives. (B) The assignments of 532 Chinese cherry accessions. The symbols and colors for each Chinese cherry accession are in agreement with those of (A). Clustering results show weak geographical signal in cultivated Chinese cherry. (C) Principle coordinate analysis of Chinese cherry accessions in WC (deep blue), CC1 (green), and CC2 (red). CC1 and CC2 are distributed in two different sides of WC without any overlapping, which indicates the separate genetic compositions between them. [file Image_4.tiff]

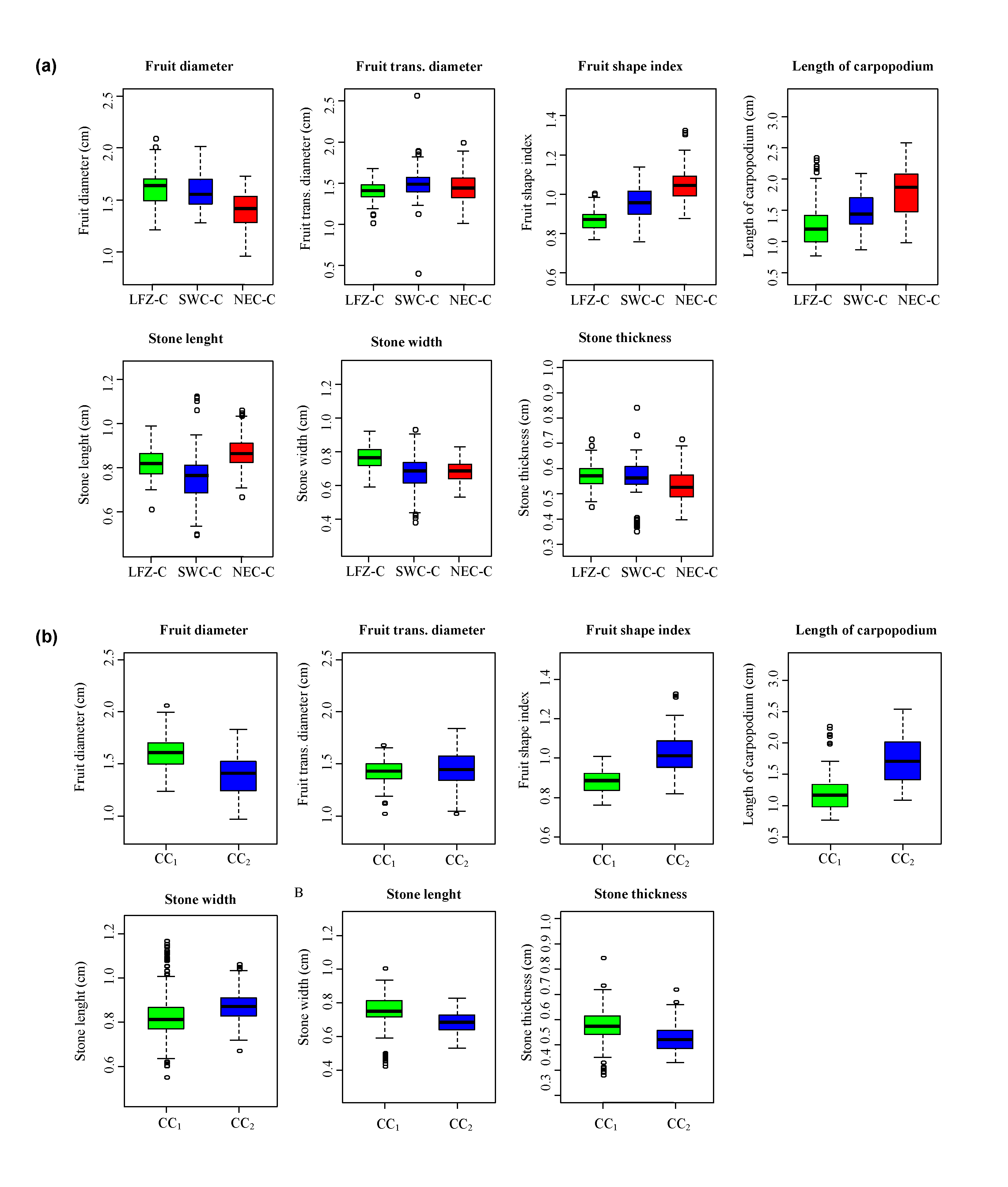

Supplement: FIGURE S5 — Box plots of seven quantitative traits of cultivated Chinese cherry accessions (A) between different geographical regions and (B) between two cultivated genetic clusters. [file Image_5.TIFF]
